# Spermatogenic cell-specific SPACA4 is essential for efficient sperm-zona pellucida binding *in vitro*

**DOI:** 10.3389/fcell.2023.1204017

**Published:** 2023-06-12

**Authors:** Lin Chen, Junli Song, Jinglei Zhang, Zicong Luo, Xuren Chen, Canquan Zhou, Xiaoting Shen

**Affiliations:** ^1^ Reproductive Center, The First Affiliated Hospital, Sun Yat-Sen University, Guangzhou, China; ^2^ Guangdong Provincial Key Laboratory for Reproductive Medicine, Guangzhou, China; ^3^ Guangdong Provincial Clinical Medical Research Center for Obstetrical and Gynecological Diseases, Guangzhou, China

**Keywords:** SPACA4, sperm, zona pellicuda, fertilization, acrosom reaction

## Abstract

Fertilization is a complex and highly regulated process that involves a series of molecular interactions between sperm and oocytes. However, the mechanisms of proteins involved in human fertilization, such as that of testis-specific SPACA4, remain poorly understood. Here we demonstrated that SPACA4 is a spermatogenic cell-specific protein. SPACA4 is expressed during spermatogenesis, upregulated in early-stage spermatids, and downregulated in elongating spermatids. SPACA4 is an intracellular protein that locates in the acrosome and is lost during the acrosome reaction. Incubation with antibodies against SPACA4 inhibited the binding of spermatozoa to zona pellucida. SPACA4 protein expression levels across different semen parameters were similar but varied significantly among patients. A prospective clinical study found no association between SPACA4 protein levels and fertilization or cleavage rates. Thus, the study suggests a novel function for SPACA4 in human fertilization in a non-dose-dependent manner. However, a larger clinical trial is required to evaluate the potential use of sperm SPACA4 protein levels to predict fertilization potential.

## 1 Introduction

Fertilization is a complex and highly regulated process that involves a series of molecular interactions between sperm and oocytes and is crucial for sexual reproduction (Chiu et al., 2014). Molecules expressed in the sperm are called egg-binding proteins, and their counterparts are termed sperm receptors. The use of transgenic mouse models has allowed researchers to identify multiple proteins that are essential for fertilization, including transmembrane protein IZUMO1 (Saito et al., 2019; Matsumura et al., 2021), transmembrane protein 95 (TMEM95) (Lamas-Toranzo et al., 2020; Inoue and Wada, 2022), sperm acrosome associated 6 (SPACA6) (Noda et al., 2020; Binner et al., 2021), the DC-STAMP-like domain-containing proteins 1 and 2 (DCST1/2) (Inoue et al., 2021; Noda et al., 2022), and the secreted protein sperm-oocyte fusion required 1 SOF1(Noda et al., 2020). The components expressed on oocytes are CD9 (Kaji et al., 2000; Miyado et al., 2000) and the glycosylphosphatidylinositol (GPI)-anchored protein JUNO (Bianchi et al., 2014; Jean et al., 2019). Notably, IZUMO1 and JUNO constitute the sole known molecular pair involved in mammalian fertilization (Bianchi et al., 2014; Grayson, 2015). However, the interaction between other proteins during fertilization remains unclear.

Bouncers, expressed in zebrafish oocytes, mediate sperm-oocyte binding and are essential for sperm entry into the oocyte (Herberg et al., 2018). Its homolog sperm acrosome associated 4 (SPACA4), on the contrary, is exclusively expressed in the male testis in internally fertilizing vertebrates and is also predicted to play a role in fertilization. Murine SPACA4 is essential for fertilization as it mediates sperm-zona pellucida binding (Fujihara et al., 2021). Furthermore, fertilization and cleavage rates were significantly reduced when boar semen was treated with anti-SPACA4 antibodies (Chen et al., 2020). Anti-SPACA4 antibodies also inhibited the binding and fusion of human sperm with zona pellucida-free hamster oocytes (Shetty et al., 2003), which was inconsistent with other findings on its role in fertilization (Fujihara et al., 2021). The present study aims to investigate the expression characteristics of *spaca4* during human spermatogenesis and in ejaculated sperm, as well as the role of SPACA4 protein in human fertilization.

## 2 Materials and methods

### 2.1 Ethical approval

This study was approved by the ethical committee of the First Affiliated Hospital of Sun Yat-sen University (No. 2021-401). Informed consent was obtained from the patients who donated their semen and immature oocytes for research purposes.

### 2.2 Secondary analyses of single-cell RNA sequencing data

Raw data from a previous study on single-cell sequencing of human testis (GSE109037) were loaded into R (version 4.2.2) and analyzed using the Seurat package (version 4.0), as previously described (Hermann et al., 2018). We excluded the data of cells expressing fewer than 200 detected genes and that of genes expressed in fewer than 3 cells. Gene expression values were log-normalized and scaled after quality control. Principal component analysis was applied to reduce dimensionality. Cluster analysis was performed, and the results were visualized using a Uniform Manifold Approximation and Projection plot. Clusters were annotated based on known gene markers (Wang et al., 2018; Salehi et al., 2021) and the expression characteristics of *spaca4* were plotted.

### 2.3 Semen and oocyte preparation

Semen samples were obtained via masturbation from patients enrolled at the study site and subjected to a direct swim-up procedure utilizing Earle Balanced Salt Solution (EBSS, Solarbio, Beijing, China) supplemented with 0.3% bovine serum albumin (BSA), 0.3 mmol/L sodium pyruvate, 0.16 mmol/L penicillin-G, 0.05 mmol/L streptomycin sulfate, and 14 mmol/L sodium bicarbonate (EBSS/0.3%BSA). Sperm were incubated in EBSS/3%BSA for 3 h to enable capacitation (Wang et al., 2021). Immature human oocytes were obtained from couples that underwent intracytoplasmic sperm injection. In the cohort study, semen samples were obtained from male patients who underwent conventional *in vitro* fertilization (IVF) programs at the study site. The liquefied semen samples were subjected to a density gradient centrifugation protocol, followed by a conventional swim-up procedure (Wang et al., 2021). Sperm in the upper medium after swim-up were collected for IVF at a concentration of 0.2 × 10^6^/mL and a proportion of the remaining sperm suspension was collected for protein analysis.

### 2.4 Immunohistochemical staining of SPACA4 in the testis

Sectioned slices of human testis from three patients with obstructive azoospermia were obtained from another project under ethical approval at the study site. Testis tissues were obtained via micro-testicular sperm extraction after patients provided written consent. The tissues were fixed in 4% paraformaldehyde, embedded in paraffin, and sectioned into slices. The slices were subjected to antigen retrieval in a microwave, followed by incubation with hydrogen peroxide for 10 min and 10% normal goat serum for 1 h at room temperature (RT). Polyclonal antibodies against human SPACA4 (ThermoFisher, Massachusetts, United States) were incubated with the slides overnight at 4°C. After washing with phosphate-buffered saline, slides were incubated with biotinylated anti-rabbit immunoglobulin G and streptavidin-peroxidase complexes (MXB, Fuzhou, China) at RT for 10 min each. Finally, the antigens were visualized by incubation with a 3,3′-diaminobenzidine solution (MXB, Fuzhou, China).

### 2.5 Immunofluorescent staining of SPACA4 on the sperm

Swim-up and unwashed sperm were fixed with 4% paraformaldehyde, smeared onto slides, and air-dried. The slides were subsequently blocked with 10% normal goat serum, with or without 0.3% Triton X-100, for 1 h at RT. After blocking, anti-SPACA4 polyclonal antibodies were added and incubated overnight at 4°C. Finally, Alexa Fluor^®^ 555 conjugated anti-rabbit immunoglobulin G (Abcam, Shanghai, China) supplemented with fluorescein isothiocyanate-conjugated lectin from *Pisum sativum* (25 ug/mL, FITC-PSA, Sigma, CA, United States) was used to visualize the antigens. The slides were examined under a fluorescence microscope (Olympus).

### 2.6 Western blotting analysis

Raw protein from the sperm was extracted using a 1% SDS solution. Aliquots of 20 μg of whole cell extracts were resolved by 15% SDS-PAGE and transferred onto a polyvinylidene difluoride membrane. After blocking with a fast-blocking buffer (EpiZyme, Shanghai, China) for 15 min at RT, the membrane was incubated with anti-SPACA4 antibodies at 4°C overnight, followed by incubation with horseradish peroxidase-conjugated secondary antibodies (proteintech, Wuhan, China) at RT for 1 h. The bands were visualized using a chemiluminescence kit (EpiZyme, Shanghai, China).

### 2.7 Measurement of sperm viability

Flow cytometry was used to determine sperm viability as previously described (Da Costa et al., 2021). Briefly, sperm suspensions were incubated with propidium iodide (Biosharp, Heifei, China) for 5 min at RT in the dark, and sperm viability was examined using a flow cytometer (CytoFlex, Beckman). The data were processed using FlowJo software (version 10.8.1).

### 2.8 Measurement of sperm motility

Sperm motility was evaluated using a computer-assisted semen analysis system, in which each measurement was taken using a prewarmed microscope platform. At least 500 sperm cells per sample were randomly selected and evaluated for the following parameters, as described in (Wang et al., 2021): 1) progressive motile (PR, %) sperm; 2) non-progressive motile (NPR, %) sperm; 3) immotile (IM, %) sperm; 4) curvilinear velocity (VCL, μm/s); 5) straight-line velocity (VSL, μm/s); 6) average path velocity (VAP, μm/s); 7) mean linearity (LIN, VSL/VCL, %); 8) straightness (STR, VSL/VAP, %); WOB (wobble, VAP/VCL, %); 10) amplitude of lateral head displacement (ALH, μm) and 11) beat-cross frequency (BCF, Hz).

### 2.9 Determination of calcium ionophore induced-acrosome reaction

The acrosome reaction was induced by incubating capacitated sperm with 2.5 μM calcium ionophore A23187 at 37°C for 1 h. The acrosomal status was assessed by immunostaining using FITC-PSA (Sigma, St. Louis, United States) as described (Efrat et al., 2019). Briefly, the sperm suspension was air-dried on slides and fixed with 95% ethanol before incubation with FITC-PSA (25 μg/mL) for 30 min. Acrosome-reacted sperm was defined as the reduction of FITC-PSA fluorescence intensity in the acrosome region. At least 200 sperm cells were evaluated per sample under a fluorescence microscope.

### 2.10 Hemizona binding assay

The hemizona-binding assay was conducted as previously described (Yao et al., 1998; Wang, et al., 2021), with some modifications. Immature oocytes were bisected into two identical hemizona, and each hemizona was separately incubated with a sperm suspension of concentration 4 × 10^5^ sperm/mL in a 20 µl droplet covered with prewarmed mineral oil under standard culture conditions of 37°C and 5% CO_2_ for 3 h. The hemizona were then gently washed several times to remove loosely bound sperm and fixed onto a slide. After washing with PBS, slides were incubated with a DAPI solution. The number of sperm cells bound to the hemizona was subsequently evaluated using a fluorescent microscope. The results were reported as hemizona index (HZI), which was calculated by dividing the number of sperm bound to the study hemizona by that bound to the control hemizona.

### 2.11 Ovarian stimulation and *in vitro* fertilization

Couples undergoing conventional IVF were recruited to investigate the relationship between sperm SPACA4 expression and IVF outcomes. The inclusion criterion was as follows: 1) couples with a female partner of age ≤40 years and oocyte yield of ≥10. The exclusion criteria were as follows: 1) couples undergoing IVF with frozen-thawed oocytes, 2) couples with known fertilization failure, 3) couples with the female partner carrying ovum with abnormal zona pellucida, and 4) couples with the female partner diagnosed with ovarian diseases affecting oocyte quality, such as endometriosis. Ovarian hyperstimulation was performed using either the conventional gonadotropin-releasing hormone (GnRH) agonist long protocol or the GnRH antagonist protocol, as previously described (Yang et al., 2021). Subsequently, the oocytes were incubated with 2 × 10^5^/mL sperm suspension under mineral oil and observed under a microscope 16–17 h after insemination. Normal fertilization was defined as the presence of two pronuclei (2PN).

### 2.12 Statistical analysis

Normally distributed data are presented as mean ± standard deviation (SD). Comparisons between two or more groups were performed using Student’s *t*-test or one-way analysis of variance. In contrast, non-normally distributed data are expressed as median (interquartile range, IQR) and compared using the Mann-Whitney *U* test or Kruskal-Wallis test. The *p*-values were adjusted using Bonferroni’s *post hoc* test. The correlation between sperm SPACA4 protein levels and IVF outcomes was assessed using Spearman’s rank coefficient. Statistical significance was set at *p*-value < 0.05.

## 3 Results

### 3.1 *spaca4* is expressed exclusively in germ cells

**FIGURE 1:**
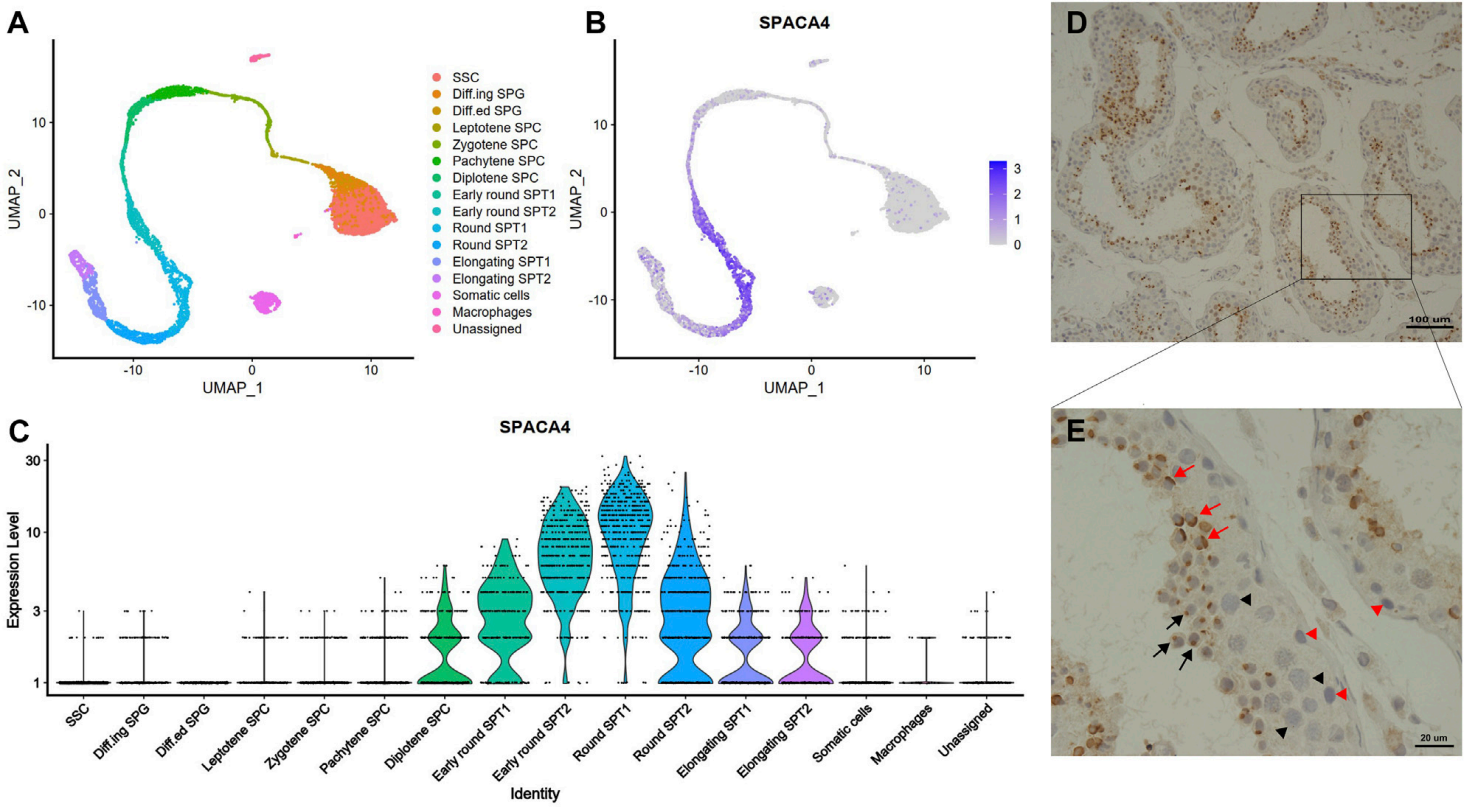
The expression profiles of SPACA4 in human testis. **(A)** Overview of the sequential stages of human spermatogenesis detected via single-cell RNA sequencing derived from published single-cell RNA-Seq data, represented in a UMAP1-UMAP2 (Uniform Manifold Approximation and Projection) plot; **(B,C)** Expression patterns and values of *spaca4* during human spermatogenesis. **(D,E)** Expression patterns of SPACA4 during human spermatogenesis analyzed by immunohistochemistry. Black and red arrows indicate SPACA4 expression during the Golgi and cap phases of acrosomal morphogenesis, respectively. Arrow, spermatids; black triangle, primary spermatocytes; red triangle, spermatogonial stem cells. SSC, spermatogonial stem cells; SPG, spermatogonia; SPC, spermatocyte; SPT, spermatid.

The results from the secondary analysis of single-cell RNA sequencing data revealed that *spaca4* was specifically expressed in spermatogenic cells and upregulated during spermiogenesis (Figures 1A–C). The transcription of *spaca4* was detected from the diplotene phase of primary spermatocyte development, and it was significantly upregulated in the early stages of round spermatids but downregulated in elongating spermatids. In contrast, *spaca4* was barely expressed in other cells, including spermatogonial stem cells (SSC), spermatogonia (SPG), Sertoli cells, Leydig cells, and macrophages.

To validate the scRNA sequencing results, we explored the expression of SPACA4 protein in human testis with normal spermatogenesis using immunohistochemical staining. As expected, SPACA4 was exclusively detected in spermatids (Figure 1D) and was located on one side of the cell in the shape of a dot (Figure 1E, marked by a black arrow) or a cap (Figure 1E, marked by a red arrow), which resembled the appearance of the acrosome in the Golgi and cap phases, respectively. SPACA4 protein was not observed in SSC, primary spermatocytes, or somatic cells.

### 3.2 SPACA4 experiences loss during acrosome reaction in ejaculated sperm

**FIGURE 2:**
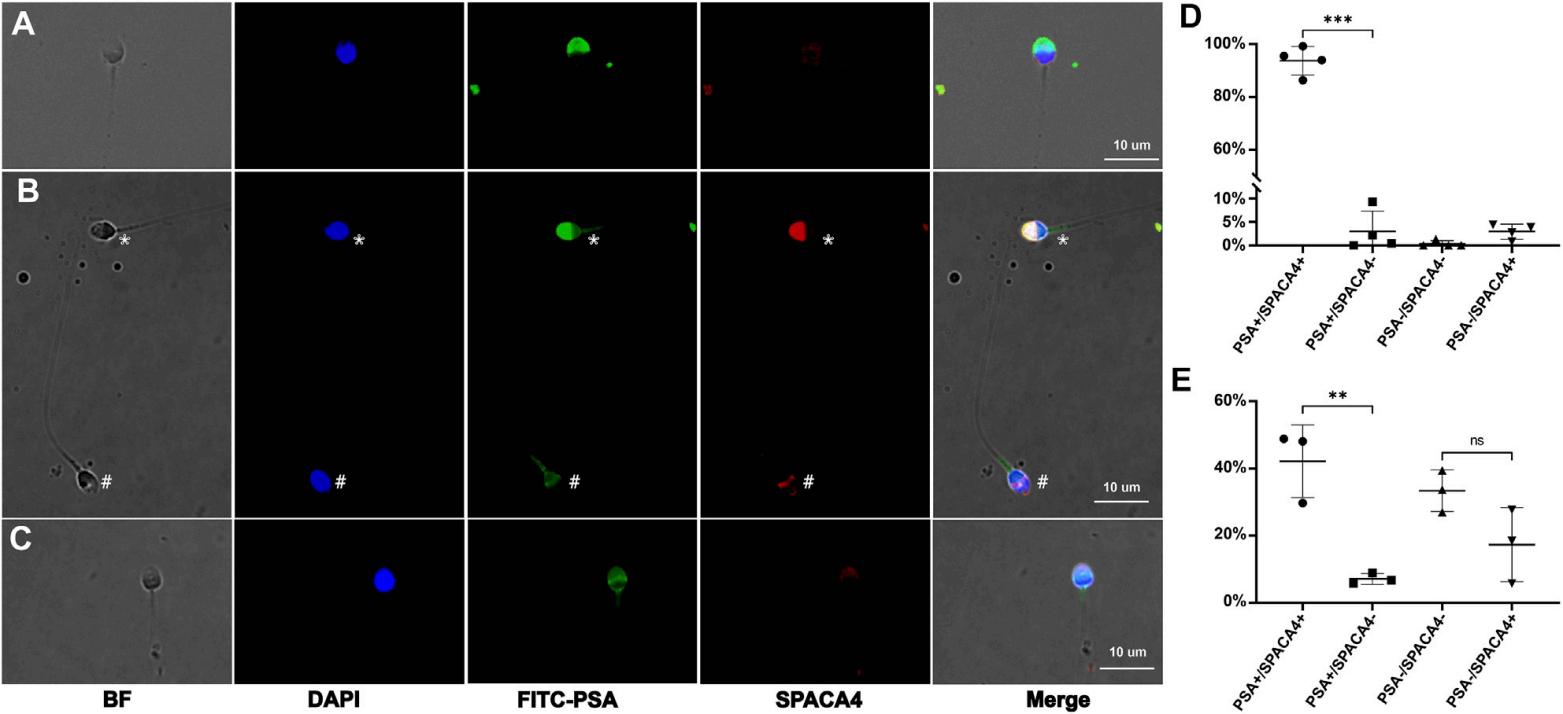
The expression profiles of human SPACA4 in ejaculated sperm. Immunofluorescence staining of acrosome (green) and SPACA4 (red) under non-permeabilizing **(A)** and permeabilizing conditions **(B)**. SPACA4 was located in the whole acrosome region [**(B)**, marked by an asterisk], but the fluorescence dimmed as the acrosome reaction proceeded **(C)**. In acrosome-reacted sperm, the fluorescence of SPACA4 was confined to the equatorial segment [**(B)**, marked by a pound]. The fluorescence patterns of FITC-PSA and SPACA4 in uncapacitated **(D)** and acrosome-reacted **(E)** sperm. **p < 0.01, ***p < 0.001.

To investigate the expression characteristics of SPACA4 protein in ejaculated sperm, we performed immunofluorescence staining of swim-up and unwashed sperm from at least three semen samples in each group. SPACA4 was detected under permeabilizing conditions (Figure 2B, marked by an asterisk) but not under non-permeabilizing conditions (Figure 2A). In addition, we noticed that the fluorescence of SPACA4 dimmed as the acrosome reaction proceeded. SPACA4 was located in the whole acrosome region in acrosome-intact sperm (Figure 2B, marked by an asterisk), but it turned dim as the acrosome reaction began (Figure 2C). In acrosome-reacted sperm, SPACA4 was confined to the equatorial segment (Figure 2B, marked by a pound). In some cases, SPACA4 was undetectable in acrosome-reacted sperm. In acrosome-intact (unified and intense FITC-PSA fluorescence signal) sperm, 96.87% of sperm were SPACA4 positive (Figure 2D). After acrosome reaction (reduced FITC-PSA fluorescence signal), however, only 32.81% of sperm were SPACA4 positive in the sperm head, 67.19% of sperm experienced SPACA4 loss after acrosome reaction (Figure 2E, *p*-value = 0.074).

### 3.3 Anti-SPACA4 antibodies inhibited sperm-zona pellucida binding

**FIGURE 3:**
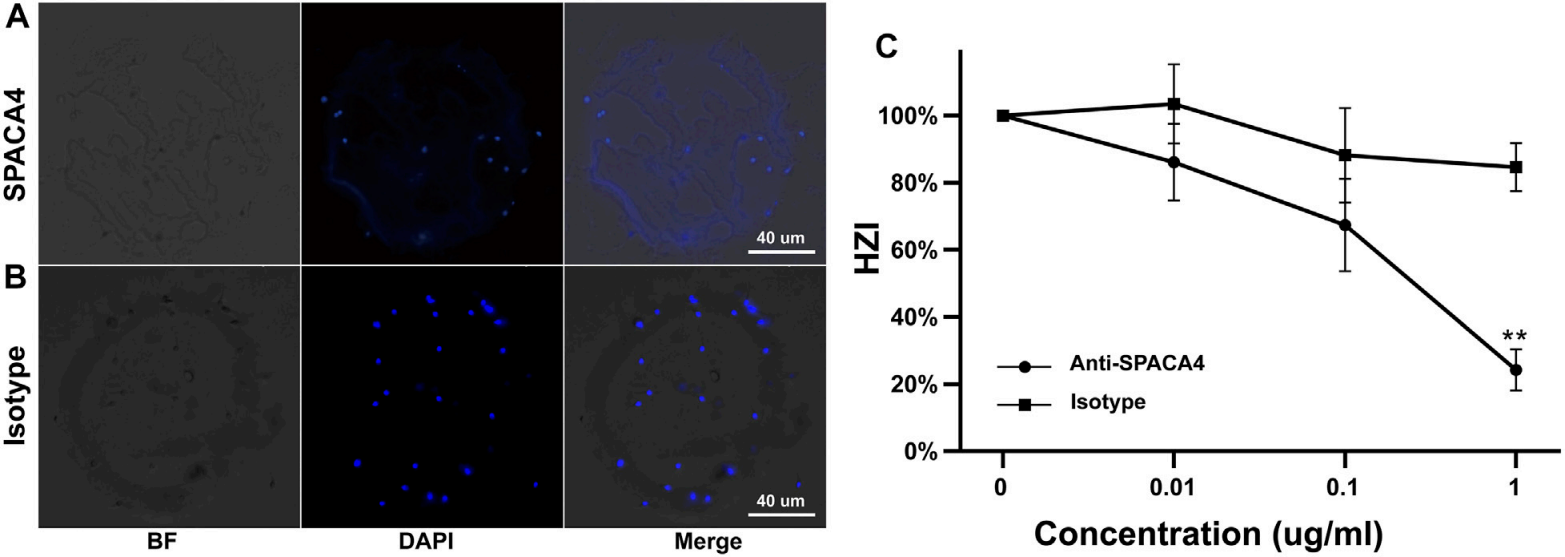
Inhibition of the binding of capacitated sperm to hemizona by anti-SPACA4 antibodies. Capacitated sperm were pretreated with anti-SPACA4 antibodies **(A)** or pre-immune rabbit immunoglobulin G [Isotype, **(B)**] before incubation with paired hemizona (*n* = 5). Antibodies against SPACA4 inhibited the number of sperm bound to hemizona **(C)**. **p < 0.01.

Preincubation with anti-SPACA4 antibodies at a concentration of 1 ug/mL significantly (*p* < 0.05) reduced the number of sperm that bound to hemizona (Figure 3A) by approximately 60% compared with that when incubated with pre-immune rabbit immunoglobulin G (Figures 3B,C). However, the antibodies did not affect the viability of capacitated and acrosome-reacted sperm (Supplementary Table S1), sperm motility (Supplementary Table S2), or the calcium ionophore-induced acrosome reaction rates (Supplementary Table S3).

### 3.4 SPACA4 expression varies significantly between individuals

**FIGURE 4:**
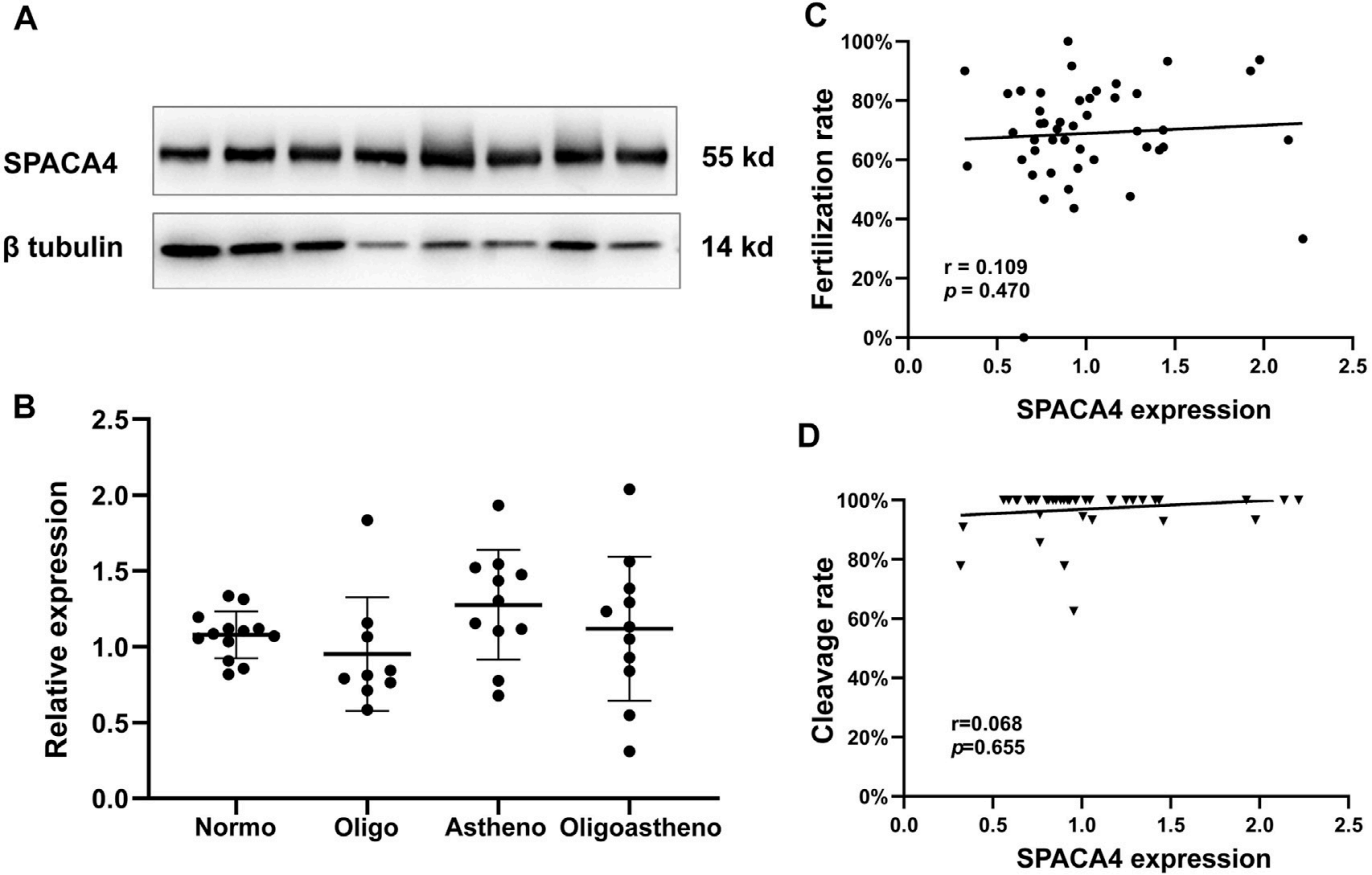
Expression of SPACA4 in ejaculated sperm and the relationship between SPACA4 expression and IVF outcomes. **(A)** The expression of sperm SPACA4 varied between individuals; **(B)** the expression of sperm SPACA4 between different semen samples was similar (*p* > 0.05); **(C,D)** the protein levels of sperm SPACA4 were not significantly correlated with fertilization or cleavage rates in IVF. Normo, Normozoospermia; Oligo, oligospermia; Astheno, asthenospermia; Oligoastheno, oligoasthenospermia.

We explored the relationship between semen parameters and the expression levels of sperm SPACA4 protein. Semen samples from normozoospermic (*N* = 13), oligospermic (*N* = 9), asthenospermic (*N* = 11), and oligoasthenospermic (*N* = 11) patients were collected, and the relative expression of SPACA4 was determined by Western blotting, which varied significantly between individuals (Figure 4A). However, the overall expression levels between different semen parameters were similar (*p*-value > 0.05) (Figure 4B).

### 3.5 SPACA4 expression was not associated with fertilization rates

Next, we investigated whether SPACA4 expression could predict the fertilization potential. Thus, couples undergoing the conventional IVF program at the study site were recruited to determine whether the upregulated SPACA4 expression was correlated with favorable fertilization outcomes. Forty-six couples were enrolled in the study. Baseline information is shown in Table 1, and IVF outcomes are presented in Table 2. The Spearman correlation coefficient between SPACA4 expression and fertilization rate was 0.109 (*p* = 0.470) (Figure 4C), and the Spearman correlation coefficient between SPACA4 expression and cleavage rate was 0.068 (*p* = 0.655) (Figure 4D), indicating no significant correlation between sperm SPACA4 protein levels and fertilization or cleavage rates.

**TABLE 1:** Baseline characteristics of the enrolled participants in the cohort study.

	Overall (*n* = 46)	Low (*n* = 23)	High (*n* = 23)	*p*-value
Maternal age (y)	30.74 ± 3.83	31.22 ± 4.1	30.26 ± 3.56	0.403
Paternal age (y)	32.20 ± 4.05	32.00 ± 4.60	32.39 ± 3.50	0.747
COS protocol				0.369
GnRH-agonist (*n*, %)	19 (41.30)	8 (17.40)	11 (23.90)	
GnRH-antagonist (*n*, %)	27 (58.70)	15 (32.60)	12 (26.10)	
Baseline hormone levels				
FSH (IU/L)	5.76 ± 1.38	5.72 ± 1.23	5.76 ± 1.38	0.920
LH (IU/L)	3.88 (2.38)	2.92 (1.57)	3.88 (2.38)	0.238
E2 (pg/mL)	29.00 (11.00)	30.00 (14.00)	29.00 (11.00)	1.000
AMH (ng/mL)	5.28 (5.08)	4.59 (4.06)	5.28 (5.08)	0.298
Semen parameters				
Volume (mL)	2.00 (1.30)	3.00 (1.50)	2.00 (1.30)	0.018
Sperm concentration (×10^6^/mL)	61.60 (52.2)	91.10 (46.10)	61.60 (52.2)	0.077
PR (%)	37.56 ± 10.26	44.35 ± 14.65	37.56 ± 10.26	0.080
SPACA4 expression	0.92 (0.52)	0.74 (0.20)	1.25 (0.43)	<0.001
Number of oocytes	17.00 (10.50)	17.00 (11.00)	16.00 (10.00)	1.000

COS, controlled ovarian stimulation; GnRH, gonadotropin-releasing hormone; PR: progressive motile.

**TABLE 2:** IVF outcomes observed in the study.

	Overall (*n* = 46)	Low (*n* = 23)	High (*n* = 23)	*p*-value
Fertilization rate (%)	69.85 (22.35)	69.23 (24.46)	70.00 (19.02)	1.000
Cleavage rate (%)	100.00 (0)	100.00 (1.19)	100.00 (0)	—

## 4 Discussion

SPACA4, also termed sperm acrosomal membrane-associated protein 14 (SAMP14), is a testis-specific protein in internally fertilizing vertebrates (Shetty et al., 2003; Herberg et al., 2018). However, its expression characteristics at the single-cell level remain unclear. In the current study, we explored the expression characteristics of *spaca4* using published single-cell RNA sequencing data of human spermatogenesis (Hermann et al., 2018), demonstrating that *spaca4* is exclusively expressed in spermatogenic cells and not in other somatic cells in the testis. In addition, *spaca4* is upregulated during the early stages of spermiogenesis and downregulated in elongated spermatids. Immunohistochemical staining of the testis confirmed that SPACA4 is located on one side of the plasma membrane of spermatids in a dot or cap shape, which resembles the appearance of the acrosome in the Golgi or cap phases of spermiogenesis, respectively (Ito and Toshimori, 2016). These findings indicated that SPACA4 is expressed during acrosomal morphogenesis. In contrast to SPACA1 (Fujihara et al., 2012), the loss of SPACA4 does not affect sperm morphology in mice (Fujihara et al., 2021), suggesting that SPACA4 may not be involved in sperm morphology transformation during spermiogenesis. However, there is no report of mutations in *spaca4* in humans. Malcher et al. (2013) found that *spaca4* is one of the most significantly downregulated (fold change = 5.71) genes in patients with idiopathic non-obstructive azoospermia compared with those in control patients. This indicates that *spaca4* may serve as a potential biomarker of azoospermia and as a molecular indicator that could determine the particular stage of impaired spermatogenesis.

Immunoelectron microscopy located SPACA4 to the inner acrosome membrane, acrosome matrix, and outer acrosome membranes. While it was also detected in immunofluorescence assay under non-permeabilized conditions, this may be attributed to membrane damage during washing by centrifuge (Shetty et al., 2003). Recent predictions from the Alliance of Genome Resources database indicate that SPACA4 is also located in the extracellular region and plasma membrane^1^. But our findings confirmed the intracellular distribution of human SPACA4 as polyclonal antibodies against it detected immunofluorescence under permeabilized conditions but not under non-permeabilized conditions. Besides, in contrast to murine SPACA4, which does not re-localize after acrosome reaction (Fujihara et al., 2021), a gradual loss of SPACA4 was observed in a proportion of sperm during the acrosome reaction in humans, with the remaining SPACA4 restricted to the equatorial segment. In the remaining sperm, SPACA4 was still retained in the equatorial segment or the acrosome region after acrosome reaction. The acrosome reaction is an exocytotic process that includes the fusion and release of plasma and the outer acrosomal membrane (Patrat et al., 2000), leading to the exposure of the inner acrosomal membrane. During this process, SPACA4 located on the outer acrosomal membrane, and acrosomal matrix was released, resulting in a decline in fluorescence intensity. PGAP6, a phospholipase A2 with specificity to glycosylphosphatidylinositol, has narrow specificity toward various GPI-anchored proteins, including SPACA4 (Lee et al., 2020). It is also expressed in human sperm; however, whether the release of SPACA4 in the inner acrosomal membrane during acrosome reaction depends on PGAP6 remains to be investigated. In addition, the action of sperm cells with different SPACA4 expression patterns after the acrosome reaction in fertilization is currently unclear.

Penetrating the extracellular matrix of the cumulus, oocyte-binding proteins on the surface of the sperm interact with zona pellucida glycoproteins (primary zona pellucida binding). After the acrosome reaction, which is induced by ZP3 in humans (Chiu et al., 2008), proteins on the inner acrosomal membrane bind to the zona pellucida (secondary zona pellucida binding) to maintain sperm adherence to ensure that the sperm can penetrate the zona pellucida and reach the perivitelline space. SPACA4 is located on the inner acrosomal membrane (Shetty et al., 2003), thus, it may be involved in the secondary zona pellucida binding instead of primary zona pellucida binding. In the subsequent process of fertilization, the plasma membrane of the equatorial segment of the sperm head, where SPACA4 is present after the acrosome reaction (Figure 2B), fuses with the oocyte membrane to form a zygote (Familiari et al., 2006). Therefore, anti-SPACA4 antibodies can inhibit the binding and fusion of human sperm to ZP-free hamster oocytes (Shetty et al., 2003). Chen et al. (2020) found that treating boar semen with antibodies against SPACA4 significantly reduced fertilization and cleavage rates. Similarly, Fujihara et al. (2021) observed that the natural fertility of *spaca4* knockout mice was significantly compromised. Sperm from homozygous (*spaca4* ^*−/−*^) mice lead to severely reduced fertilization rates compared with those from wild-type (*spaca4* ^*+/+*^) or heterozygous (*spaca4* ^*+/−*^) mice (2.9% vs. 59.4% vs. 33.4%). However, the underlying mechanism was binding failure between the sperm and the zona pellucida. Murine sperm lacking SPACA4 had a comparable potential to fertilize zona pellucida-free oocytes. Due to ethical considerations, the incubation of human sperm samples with anti-SPACA4 antibodies to analyze their effect on human reproductive performance is not allowed. Therefore, the hemizona assay was performed to investigate the role of SPACA4 in human fertilization. Our results found that preincubation with 1 μg/mL anti-SPACA4 antibodies significantly reduced the number of sperm bound to hemizona by approximately 60%. Under natural conditions, sperm bind to the zona pellucida to prepare for fertilization. Thus, it can be concluded that SPACA4 plays a role in sperm zona pellucida recognition and binding during fertilization.

According to (Chen et al., 2020), the protein levels of sperm SPACA4 in high-fertility boar were significantly higher than those in low-fertility boar. In addition, SPACA4 protein levels were positively correlated with boar reproductive performance, including sows’ farrowing rate and reproductive efficiency, although not correlated with litter size. In the current study, we observed different SPACA4 protein expression levels in ejaculated sperm between individual patients; however, whether this variation correlates with reproductive performance remains unknown. Therefore, couples undergoing conventional IVF programs were recruited to investigate this relationship. To minimize random errors, couples were included only when the number of oocytes retrieved was ≥10, and couples with diseases that may affect fertilization were excluded. The sperm used for fertilization and SPACA4 protein quantification was obtained from the same semen sample, which increased the reliability of the analysis. The semen volume was significantly higher in the “High group” but all oocytes were inseminated at a concentration of 2 × 10^5^ sperm/mL, eliminating potential bias. However, our results suggest that sperm SPACA4 protein levels were not significantly correlated with fertilization or cleavage rates. This finding, together with the inhibitory effect of the anti-SPACA4 antibodies on sperm-zona pellucida binding, suggests that SPACA4 acts as an important protein in fertilization, but in a non-dose-dependent manner. This means the presence of SPACA4 is significant for efficient sperm-zona pellucida binding, the preconditions of fertilization, but its accumulation above a certain threshold contributes little to fertilization in humans, showing different values of sperm SPACA4 protein in predicting reproductive performance between human and boar (Chen et al., 2020). There are at least three possible explanations for the discrepancies. First, species specificities might make some contributions. Second, the primary reproductive outcomes used to represent reproductive performance in this study were different from those in the boar study (Chen et al., 2020). Third, unlike intracellular sperm injection procedures, denudation and maturity assessment of oocytes are not conducted in conventional IVF procedures (Van de Velde et al., 1998). Certain immature oocytes would not be fertilized, leading to the underestimation of true fertilization rates in such cases, which is a source of bias. In the cohort study, one couple experienced complete fertilization failure, and the relative expression level of sperm SPACA4 was 0.65 (normalized using the levels of beta-tubulin protein), just below the median SPACA4 level (0.92). Hence, the fertilization failure should be attributed to other factors.

This study was limited by the failure to identify the receptor on the zona pellucida that interacts with SPACA4. In addition, oocyte maturity was not evaluated during IVF, making immature oocytes a possible source of bias in fertilization rates. Furthermore, reproductive outcomes were not evaluated due to embryo cryopreservation.

## 5 Conclusion

*spaca4* is a spermatogenic cell-specific gene that is initially upregulated and subsequently downregulated during the late stages of spermatogenesis (spermiogenesis stages). SPACA4 is an intracellular protein and it is lost during the acrosome reaction. SPACA4 is essential for effective sperm-zona pellucida binding during fertilization but its protein levels do not correlate with fertilization and cleavage rates in IVF procedures.

## Data Availability

The original contributions presented in the study are included in the article/Supplementary Material, further inquiries can be directed to the corresponding authors.

## Footnotes

1
https://www.alliancegenome.org/gene/HGNC:16441
