# Supplementary material for: Spermatogenic cell-specific SPACA4 is essential for efficient sperm-zona pellucida binding in vitro
Source: Front Cell Dev Biol. 2023 Jun 12;11:1204017. doi: 10.3389/fcell.2023.1204017 (PMC10291262; doi:10.3389/fcell.2023.1204017)
Supplement: Supplementary file 1 [file Table1.docx]

| **Supplementary Table S1. Effects of anti-SPACA4 antibodies on sperm viability at different stages under different concentrations** | | | |
| --- | --- | --- | --- |
| **Treatment** | **Concentration**  **(ug/ml)** | **Capacitation stage** | **Acrosome reaction stage** |
| Blank control* | - | 86.56±4.59 | 85.83±3.80 |
| Blank control | - | 85.52±3.33 | 80.56±5.63 |
| Isotype | 1 | 84.18±3.70 | 81.64±4.29 |
|  | 0.1 | 84.52±3.49 | 81.88±2.91 |
|  | 0.01 | 85.65±3.54 | 81.90±3.88 |
| SPACA4 antibody | 1 | 85.20±2.68 | 81.86±3.70 |
|  | 0.1 | 85.07±2.97 | 82.24±3.98 |
|  | 0.01 | 84.87±3.20 | 81.90±4.47 |
| *, sperm viability measured before antibody treatment. | | | |

At the capacitation stage, Swim-up sperm were incubated in EBSS/3%BSA in the presence of anti-SPACA4 antibodies at 37℃ for 1 hour before viability measurement. At the acrosome stage, capacitated sperm were incubated in EBSS supplemented with calcium ionophore at 2.5uM for 1 hour in the presence of antibodies. Sperm viability was determined by propidium iodide staining and flow cytometry before and after antibody treatment. Data represent the mean ± SD (N=5).

Sperm motility was measured by a computer-assisted semen analysis system. Data represent the mean ± SD (N=3).

| **Supplementary Table S2. Effects of anti-SPACA4 antibody on sperm motility.** | | | | | | | | | | | | |
| --- | --- | --- | --- | --- | --- | --- | --- | --- | --- | --- | --- | --- |
| **Treatment** | **Concentration**  **(μg/mL)** | **PR motile**  **(%)** | **NPR**  **motile (%)** | **Immotile**  **(%)** | **VCL**  **(μm/s)** | **VSL**  **(μm/s)** | **VAP**  **(μm/s)** | **LIN**  **(%)** | **STR**  **(%)** | **WOB**  **(%)** | **ALH**  **(μm)** | **BCF**  **(Hz)** |
| Blank control* | - | 89.33±2.45 | 7.17±1.66 | 3.50±1.21 | 82.00±1.39 | 21.10±0.35 | 39.8±1.38 | 27.23 ± 1.5 | 53.27 ± 0.29 | 50.10 ± 3.12 | 4.10 ± 0.17 | 6.33 ± 0.81 |
| Blank control | - | 84.10±3.18 | 10.8±0.56 | 5.10±2.72 | 84.17±5.92 | 21.37±1.57 | 39.5±0.78 | 26.00 ± 3.60 | 52.73 ± 4.26 | 47.67 ± 2.90 | 4.23 ± 0.31 | 5.33 ± 0.31 |
| Isotype | 1 | 74.35±7.14 | 17.90±4.38 | 7.75±2.76 | 71.67±8.37 | 20.33±3.15 | 35.5±4.67 | 29.27± 1.94 | 55.73 ± 2.49 | 50.43 ± 1.88 | 3.60 ± 0.36 | 5.7 ± 0.78 |
|  | 0.1 | 79.27±6.86 | 14.27±5.38 | 6.47±2.95 | 74.40±6.22 | 20.25±4.60 | 36.55±4.46 | 27.80 ± 3.39 | 53.35 ± 5.02 | 49.95 ± 1.20 | 3.80 ± 0.28 | 5.2 ± 0.85 |
|  | 0.01 | 82.17±4.77 | 12.10±3.22 | 5.73±2.01 | 75.35±3.89 | 18.67±1.95 | 34.47±3.95 | 27.73 ± 2.85 | 52.03 ± 2.71 | 51.67 ± 4.39 | 3.50 ± 0.62 | 5.33 ± 0.61 |
| SPACA4 antibody | 1 | 80.97±3.06 | 13.30±2.38 | 5.73±0.91 | 77.2±6.86 | 21.53±1.01 | 38.13±2.21 | 28.60 ± 1.85 | 54.93 ± 2.12 | 50.67 ± 2.28 | 3.90 ± 0.35 | 5.60 ± 0.3 |
|  | 0.1 | 79.30±5.37 | 15.03±2.85 | 5.67±2.57 | 71.57±2.32 | 20.93±2.18 | 35.97±1.38 | 30.07 ± 2.84 | 56.40 ± 4.03 | 51.30 ± 1.40 | 3.67 ± 0.12 | 5.6 ± 0.61 |
|  | 0.01 | 85.47±0.86 | 11.43±0.72 | 3.10±1.51 | 74.27±6.06 | 20.57±1.21 | 36.57±1.76 | 28.93 ± 1.97 | 54.90 ± 2.09 | 50.83 ± 1.46 | 3.733 ± 0.32 | 5.67 ± 0.25 |
| *, sperm motility measured before antibody treatment. | | | | | | | | | | | | |

| **Supplementary Table S3. Effects of anti-SPACA4 antibody on calcium ionophore-induced acrosome reaction rates.** | | |
| --- | --- | --- |
| **Treatment** | **Concentration**  **(μg/mL)** | **Acrosome reaction rate** |
| Blank control | - | 48.66±5.00 |
| Isotype | 1 | 45.88±4.56 |
|  | 0.1 | 44.89±4.68 |
|  | 0.01 | 48.90±2.91 |
| SPACA4 antibody | 1 | 46.29±3.56 |
|  | 0.1 | 44.19±4.31 |
|  | 0.01 | 48.80±1.52 |

Capacitated sperm were incubated with calcium ionophore A23187 at 2.5 uM in the presence of anti-SPACA4 antibodies at 37℃ for 1 hour. Sperm acrosome reaction was determined by FITC-PSA staining. Data represent the mean ± SD (N=4).
